# Diffusion Kurtosis Imaging of Substantia Nigra Is a Sensitive Method for Early Diagnosis and Disease Evaluation in Parkinson's Disease

**DOI:** 10.1155/2015/207624

**Published:** 2015-12-03

**Authors:** Guohua Zhang, Yuhu Zhang, Chengguo Zhang, Yukai Wang, Guixian Ma, Kun Nie, Haiqun Xie, Jianping Liu, Lijuan Wang

**Affiliations:** ^1^Southern Medical University, Guangzhou, Guangdong 510515, China; ^2^Department of Neurology, Guangdong Neuroscience Institute, Guangdong General Hospital, Guangdong Academy of Medical Sciences, Guangzhou, Guangdong 510080, China; ^3^Department of Neurology, The First People's Hospital of Foshan, Foshan, Guangdong 528000, China

## Abstract

*Background.* To diagnose Parkinson disease (PD) in an early stage and accurately evaluate severity, it is important to develop a sensitive method for detecting structural changes in the substantia nigra (SN).* Method.* Seventy-two untreated patients with early PD and 72 healthy controls underwent diffusion tensor and diffusion kurtosis imaging. Regions of interest were drawn in the rostral, middle, and caudal SN by two blinded and independent raters. Mean kurtosis (MK) and fractional anisotropy in the SN were compared between the groups. Receiver operating characteristic (ROC) and Spearman correlation analyses were used to compare the diagnostic accuracy and correlate imaging findings with Hoehn-Yahr (H-Y) staging and part III of the Unified Parkinson's Disease Rating Scale (UPDRS-III).* Result.* MK in the SN was increased significantly in PD patients compared with healthy controls. The area under the ROC curve was 0.976 for MK in the SN (sensitivity, 0.944; specificity, 0.917). MK in the SN had a positive correlation with H-Y staging and UPDRS-III scores.* Conclusion.* Diffusion kurtosis imaging is a sensitive method for PD diagnosis and severity evaluation. MK in the SN is a potential biomarker for imaging studies of early PD that can be widely used in clinic.

## 1. Introduction

Parkinson's disease (PD) is a neurodegenerative disease and commonly occurs in aged people. There is a selective loss of dopaminergic neurons in the substantia nigra (SN) at the point of clinical expression of PD, which results in impaired motor function and mainly includes static tremor, muscle rigidity, bradykinesia, and loss of balance. These symptoms gradually progress with time and eventually become irreversible, which seriously affects the quality of life in patients with PD [1]. In recent years, the incidence of PD has been on the rise. Most patients are not diagnosed in the early stage; thus, the optimal time to treat is lost. PD not only seriously affects the quality of life but also brings the family pain and a significant economic burden as the disease progresses [2]. For the purpose of effective control of PD, it is important to diagnose and evaluate the severity of PD as soon as possible. A study has shown that the loss of dopaminergic neurons in the SN is more apparent with the aggravation of symptoms in PD patients [3]. Therefore, finding a sensitive and noninvasive method that can detect pathologic changes of the SN* in vivo* for PD diagnosis and severity is important [4].

Diffusion tensor imaging (DTI) is a noninvasive magnetic resonance imaging (MRI) technique that can accurately and quantitatively detect the structural integrity of intracranial nerve nuclei and fiber bundles [5]. Fractional anisotropy (FA) is one of the important indices of DTI. In recent years, a number of studies have shown that FA in the SN of early-stage PD patients is decreased [6–9]. Diffusion kurtosis imaging (DKI) is an improved DTI technique and is more capable of detecting microstructural changes of tissues compared with DTI technique [10]. Mean kurtosis (MK) is a major parameter of DKI. MK values are closely related to gray matter structure. When gray matter structures are more complex, MK values generated from DKI are higher [11]. Studies involving DTI and DKI for PD diagnosis in Taiwan have shown that the MK index has higher diagnostic efficiency than the FA index [12]. However, it remains unknown whether or not the phenomenon is the same in a local population. The objectives of this study were to evaluate the application of FA generated from DTI and MK generated from DKI for the diagnosis and evaluation of PD and to develop a noninvasive method for detecting structural changes of the SN in human brains with more sensitivity.

## 2. Methods

### 2.1. Participants

All examinations in this study was performed with the understanding and written consent of each subject and local ethics committee approval. Between January 2014 and December 2014, a total of 74 inpatients who attended the Department of Neurology in the First People's Hospital of Foshan were recruited into this study. All patients underwent MRI examinations and were diagnosed with early-stage PD without any treatment. Two patients were excluded because of incomplete DTI and DKI data. Seventy-two gender and age-matched healthy patients without nervous system disorders, mental illnesses, and other serious illnesses served as the control group. No brain structural abnormalities in the control group were noted on MRI.

The PD group inclusion criteria were as follows: (1) being >50 years of age, (2) meeting the UK Parkinson's Disease Society Brain Bank Clinical Diagnostic Criteria, and (3) being with Hoehn and Yahr (H-Y) stages 1-2. The exclusion criteria were as follows: (1) secondary Parkinson syndrome and Parkinsonism-Plus, (2) cardiac insufficiency or severe hepatic and renal impairment, (3) communication barrier, (4) being unable to complete the MRI, and (5) poor compliance.

Demographics, such as age, gender, education level, and occupation, were obtained from all participants and recorded. All of the subjects underwent comprehensive neurologic examinations. The onset, disease duration, and evolution of the disease in patients with PD were elicited in detail. H-Y staging and the motor section of the Unified Parkinson's Disease Rating Scale (UPDRS III) [13, 14] were used to assess the severity of motor impairment.

### 2.2. Imaging

All subjects underwent MRI examinations using a 3.0 T superconducting magnetic resonance instrument (GE Signa EXCITE, GE Medical System, Waukesha, WI, USA) with an 8-channel phased array coil. T1-weighted imaging, T2-weighted imaging, FLAIR images, and diffusion-weighted imaging were assessed to exclude secondary Parkinson's syndrome caused by severe vascular disease, trauma, encephalitis, and multisystem atrophy. DTI was obtained using an echo-planar imaging technique with the following scanning parameters: repetition time/echo time, 6000/76.4 ms; motion probing gradients, 25 directions; *b* values, 0 and 1000 s/mm^2^; field of view, 240 mm^2^; matrix size, 128^2^; slice thickness, 5.0 mm with 0 mm interslice gaps; and number of excitations, 1. DKI was obtained using an echo-planar imaging technique with the following scanning parameters: repetition time/echo time, 6500/73.3 ms; motion probing gradients, 25 directions; *b* values, 0, 1000, and 2000 s/mm^2^; field of view, 240 mm^2^; matrix size, 128^2^; slice thickness, 5.0 mm with 0 mm interslice gaps; and number of excitations, 1.

All image processing operations were performed with an adw 4.5 work station function tool software. Regions of interest (ROIs) were drawn independently in the rostral, middle, and caudal SN bilaterally by two of the investigators. The method which was illustrated in the study with respect to diffusion tensor imaging in the SN of patients with PD was followed to draw the ROIs [15].

### 2.3. Statistical Analyses

Statistical analyses were carried out using the Statistical Package for the Social Sciences (version 13.0; SPSS, Inc., Chicago, IL, USA). Student's *t*-test was used to compare the indices of age, FA, and MK between the patients and the control subjects. A Chi-square test was used to compare gender differences between the groups. The FA or MK correction values were the average of FA or MK values in the rostral, middle, and caudal SN bilaterally. Unpaired *t*-tests were used to compare FA and MK in these three sections of the SN between two raters. Interrater reliability between two raters was examined using intraclass correlation coefficients. Receiver operating characteristic (ROC) curves for each index (FA and MK correction values) were used to determine the cut-off values associated with optimal sensitivity and specificity for distinguishing PD patients from healthy controls. The areas under the ROC curve (AUC) were used to compare the overall diagnostic performance of the indices (FA and MK correction values). The associations between the indices (FA and MK correction values) and disease severity were assessed by Spearman correlation coefficients. A two-tailed *P* < 0.05 was considered to be statistically significant.

## 3. Results

### 3.1. General Characteristics of Study Participants

There was no significant difference between patients and healthy controls with respect to age (*t* = 0.734, *P* = 0.464) or gender (*χ* ^2^ = 0.119, *P* = 0.731) (Table 1).

### 3.2. Comparison of FA and MK Values in the SN between the PD and Control Groups

**Figure 1:**
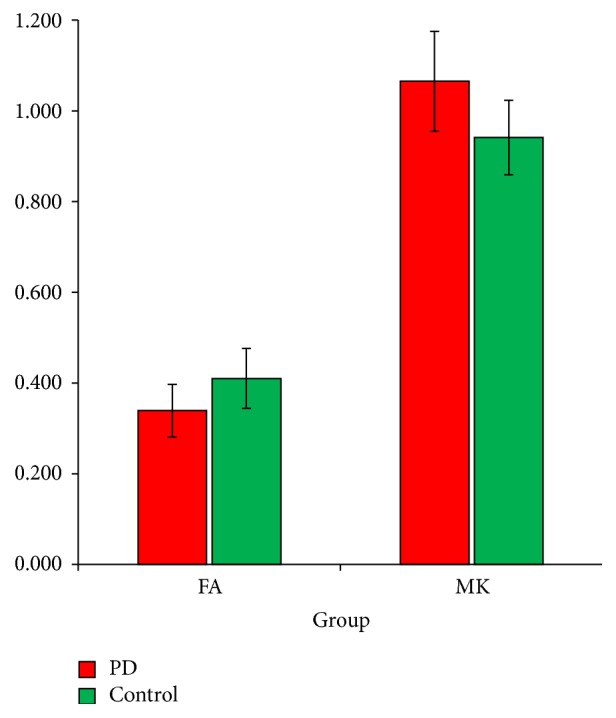
FA and MK in the SN of two groups. Mean FA and MK in bilateral rostral, middle, and caudal SN across patients with Parkinson disease (red) and control subjects (green).

Compared with healthy volunteers, FA in the SN was significantly decreased (0.339 ± 0.029 versus 0.410 ± 0.033, *t* = 13.787, *P* < 0.001) and the MK in the SN was significantly increased (1.065 ± 0.055 versus 0.941 ± 0.041, *t* = 15.400, *P* < 0.001) in the PD group. The FA and MK values in the SN of patients with PD and healthy controls are shown in Figure 1.

Unpaired *t*-tests were performed between two raters for FA and MK in the SN; both *t*-test results were nonsignificant. In the assessment of interrater reliability between raters 1 and 2, there was strong agreement for FA and MK in the SN. The intraclass correlation coefficient of FA and MK in the SN was 0.780 and 0.838, respectively.

### 3.3. ROC Curve Analyses of FA and MK in the SN

**Figure 2:**
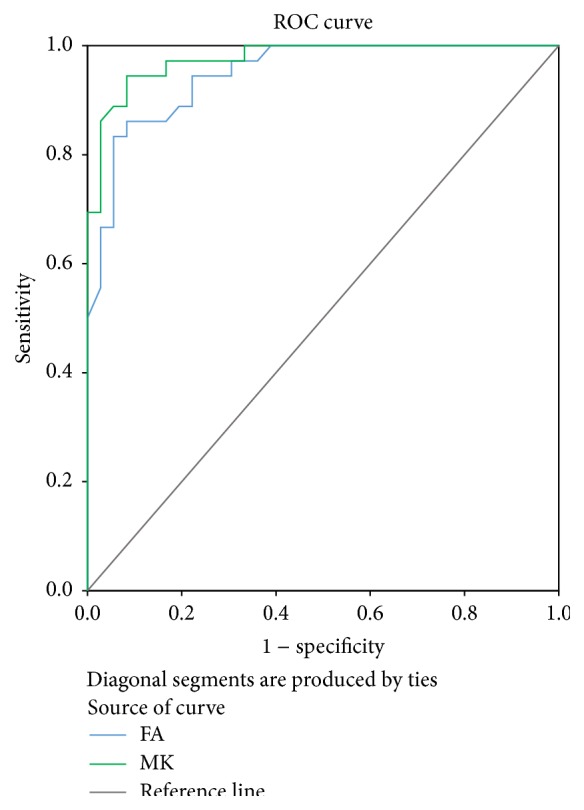
ROC analysis of FA and MK in the SN.

The AUC was 0.948 for FA in the SN (mean cut-off, 0.3805; sensitivity, 0.861; and specificity, 0.917). The AUC was 0.976 for MK in the SN (mean cut-off, 1.0000; sensitivity, 0.944; and specificity, 0.917).  Table 2 and Figure 2 show the sensitivity and specificity of FA and MK for the ROC analysis of the bilateral SN.

### 3.4. Correlation Analyses of FA and MK for the SN with H-Y Staging and UPDRS III Scores

The FA for the SN had no significant correlation with H-Y staging and UPDRS III scores (*r* = 0.011, *P* = 0.925 and *r* = 0.035, *P* = 0.774, resp.). In contrast, the MK for the SN had a positive correlation with H-Y staging and UPDRS III scores (*r* = 0.585, *P* < 0.001 and *r* = 0.700, *P* < 0.001, resp.; Table 3).

## 4. Discussion

In recent years, imaging biomarkers have become increasingly important in the diagnosis and evaluation of patients with PD [16]. Magnetic resonance diffusion imaging is one of the techniques used in the diagnosis of PD and has garnered enormous interest amongst clinicians and researchers. It is available at a moderate price with the advantages of noninvasive, accurate localization of lesions, quantification, and high repeatability [17], so magnetic resonance diffusion imaging is well suited for early diagnosis and evaluation of PD. In fact, DTI has been used in the diagnosis of PD for >10 years. In 2007, Chan et al. [6] presented a large, prospective, and case-control study involving 151 subjects (73 PD patients and 78 controls) and showed FA values of the SN in patients with PD were lower compared with controls; however, no significant differences were demonstrated for FA values of other structures. Since then, more and more studies have regarded the SN section as the most important ROI in DTI analysis for the diagnosis of PD [7, 18, 19]. In addition, some studies, which have focused on the SN bilaterally, did not show a significant difference between two laterals of the SN [20, 21]. Thus, most of the studies obtained indices for DTI of the SN bilaterally rather than unilaterally [17, 22, 23].

The most recent meta-analysis [21] concluded that the SN FA value in healthy subjects was unstable and ranged from 0.37 to 0.7. This finding was thought to be due to different study population characteristics, substantial technical differences, variation in size, placement of ROIs, and iron deposition. Therefore, the diagnosis value for PD of FA in the SN has not been established. In principal, the DKI technique makes up for some of the shortcomings of DTI. DKI can not only obtain all parameters of DTI, but also detect the nonnormal distribution diffusion of water molecules in tissues. The MK value of DKI is more valuable for diagnosing pathologic changes of isotropic structures, such as gray matter, than the FA value because of the independence on the spatial direction of structures [11]. Because the most significant pathologic changes in PD patients occur in the gray matter SN, MK values in the SN applied to the diagnosis and evaluation of PD may be more sensitive than FA values. Therefore, we compared MK and FA values in the bilateral SN of PD patients to access the application value for the diagnosis and evaluation of PD in a local population to demonstrate a safe and noninvasive method for detecting structural changes of SN in human brains with more sensitivity.

Furthermore, it has been reported that PD-related cell loss occurs mainly in the ventrolateral and caudal segment of the SN [24]. To further clarify which section of the SN in DTI has the most notable lesions with the progression of PD, investigators divided the SN into three sections (rostral, middle, and caudal SN) and then analyzed the sections. Some studies have shown that the rostral SN has the most notable lesions [25, 26], while the other studies showed that caudal SN changes most significantly [15, 20]. It still remains unclear which section should be the focus during an imaging study of the SN. A number of studies involving magnetic resonance diffusion imaging in the SN of PD patients selected the average values of the index in these three sections to analyze [23, 27]. Therefore, the mean values of MK and FA in the rostral, middle, and caudal SN were used as the corrected values for analysis to reduce the errors caused by selection of ROIs in the current study.

Our results indicated that, compared with the general population in this region, FA in the SN of PD patients was significantly decreased, which was consistent with others [6–9]. To further study the role of MK in the SN in the diagnosis of PD, DTI and DKI were performed for different cerebral ROIs in early-to-middle stage patients with PD by Wang et al. several years ago [12]. It is interesting that we cannot find any other report involving this area except Wang's study. They reported that the MK value in the SN of PD patients changed compared with healthy subjects. The MK values for the SN in the diagnosis of PD were more valuable than FA values. These results are consistent with our results. Although the results of the ROC curve analysis showed that the specificity of FA and MK in the SN is similar, the sensitivity of MK is higher than that of FA, indicating that MK in the SN is a more sensitive index for early diagnosis of PD. To show the reproducibility of the results, interrater reliability between raters 1 and 2 was examined using intraclass correlation coefficients. Because our study included patients with early-stage PD, MK in the SN may be more sensitive in the diagnosis of early-stage PD.

Meanwhile, we performed correlation analyses of FA and MK in the SN with H-Y staging and UPDRS III scores to clarify the role of MK in the evaluation of PD progression during the course of treatment. We found that FA had no significant correlation with H-Y staging and UPDRS III scores. In contrast, MK had a positive correlation with H-Y staging and UPDRS III scores. The results indicated that MK values in the SN may reflect the severity of disease; specifically, the more serious the lesions, the higher the MK values, which may promote the disease assessment of patients.

This study had several limitations. The sample size was too small to yield sufficient statistical power and the research did not involve multiple centers. Nevertheless, this was a preliminary study to confirm the potential advantages of DKI analyses.

## 5. Conclusion

In conclusion, MK in the SN of patients with early-stage PD was significantly increased. Because MK in the SN correlated with the severity of motor dysfunction in PD patients, regular MK detection may contribute to evaluate the progression of PD. As a sensitive index, MK in the SN is a potential biomarker for imaging studies of PD and may improve the diagnosis of PD.

## Figures and Tables

**Table 1:** General characteristics of study participants.

	PD group	Control group	*P* value
Gender (M/F)	26/46	28/44	0.731
Age (years)	66.83 ± 5.41	66.08 ± 6.77	0.464
Duration (months)	13.50 ± 6.79	N	
H-Y staging	1.67 (1-2)	N	
UPDRS III scores	14.94 ± 3.86	N	

**Table 2:** ROC analysis of FA and MK for the SN.

	FA	MK
Sensitivity %	86.1	94.4
Specificity %	91.7	91.7
AUC	0.948	0.976
95% confidence intervals of AUC	0.937~0.960	0.966~0.985

**Table 3:** Correlation analyses of FA and MK for the SN with H-Y staging and UPDRS III scores.

Spearman	H-Y staging	UPDRS III score
FA for the SN	*r* = 0.011, *P* = 0.925	*r* = 0.035, *P* = 0.774
MK for the SN	*r* = 0.585, *P* < 0.001	*r* = 0.700, *P* < 0.001
